# Astrocyte-secreted thrombospondin-1 modulates synapse and spine defects in the fragile X mouse model

**DOI:** 10.1186/s13041-016-0256-9

**Published:** 2016-08-02

**Authors:** Connie Cheng, Sally K. M. Lau, Laurie C. Doering

**Affiliations:** 1 McMaster Integrative Neuroscience Discovery and Study Program (MINDS), McMaster University, 1280 Main Street West, Hamilton, Ontario L8S 4L8 Canada; 2 Department of Pathology and Molecular Medicine, McMaster University, 1200 Main Street West, HSC 1R15A, Hamilton, Ontario L8N 3Z5 Canada

**Keywords:** Fragile X syndrome, Autism, Astrocytes, Secreted factor, Thrombospondin-1, Synapses, Dendritic spines

## Abstract

Astrocytes are key participants in various aspects of brain development and function, many of which are executed via secreted proteins. Defects in astrocyte signaling are implicated in neurodevelopmental disorders characterized by abnormal neural circuitry such as Fragile X syndrome (FXS). In animal models of FXS, the loss in expression of the Fragile X mental retardation 1 protein (FMRP) from astrocytes is associated with delayed dendrite maturation and improper synapse formation; however, the effect of astrocyte-derived factors on the development of neurons is not known. Thrombospondin-1 (TSP-1) is an important astrocyte-secreted protein that is involved in the regulation of spine development and synaptogenesis. In this study, we found that cultured astrocytes isolated from an *Fmr1* knockout (*Fmr1* KO) mouse model of FXS displayed a significant decrease in TSP-1 protein expression compared to the wildtype (WT) astrocytes. Correspondingly, *Fmr1* KO hippocampal neurons exhibited morphological deficits in dendritic spines and alterations in excitatory synapse formation following long-term culture. All spine and synaptic abnormalities were prevented in the presence of either astrocyte-conditioned media or a feeder layer derived from FMRP-expressing astrocytes, or following the application of exogenous TSP-1. Importantly, this work demonstrates the integral role of astrocyte-secreted signals in the establishment of neuronal communication and identifies soluble TSP-1 as a potential therapeutic target for Fragile X syndrome.

**Electronic supplementary material:**

The online version of this article (doi:10.1186/s13041-016-0256-9) contains supplementary material, which is available to authorized users.

## Introduction

Fragile X syndrome (FXS) is the most common form of intellectual disability and is a leading cause of autism spectrum disorders [1], affecting about 1/4000 males and 1/8000 females. The causative mutation for the majority of cases is a trinucleotide CGG expansion in the promoter region of the fragile X mental retardation 1 gene (*FMR1*), which induces transcriptional gene silencing and the loss of the fragile X mental retardation 1 protein (FMRP). FMRP is an RNA-binding protein that is highly involved in binding and regulating the translation, transport and stability of a subset of mRNAs to synapses [2, 3]. Fundamental research on the *Fmr1* knockout (KO) mouse has provided promising insights into the cellular and molecular underpinnings of the condition. A well-described characteristic feature of FXS is the presence of “immature” dendritic spines [4, 5]. These dendritic spine abnormalities in *Fmr1* KO mice are most pronounced during development, but also persist into adulthood [6]. As spines are thought to be the site of functional changes that mediate memory storage, an immature or otherwise aberrant morphology could represent the critical effect of the FXS mutation that underlies learning impairments.

The appropriate formation of neural connections is vastly dependent on reciprocal neuronal and glial interactions. Until recently, the majority of research into the function of FMRP, and the consequences of its absence, has largely been focused on neurons. However, it is now known that FMRP is also expressed in cells of the glial lineage [7, 8]. The expression of FMRP is typically highest in astrocytes within the first week of birth and subsequently declines to low or undetectable levels [8]. Based on these findings, work in our laboratory investigated the role of astrocytes in the development of the abnormal neurobiology of FXS. Using an astrocyte-neuron co-culture system, hippocampal neurons showed developmental delays in dendritic growth patterns and also in the expression of excitatory synapses when interfaced with astrocytes lacking FMRP [9, 10], suggesting that dysfunction in non-neuronal cells may be a contributing factor into the pathogenesis of FXS.

During development and in the mature brain, astrocytes are known to provide signals that guide synapse formation and neurite development [11–14]. Astrocytes can regulate the stability, dynamics and maturation of dendritic spines through the release of secreted factors [15, 16]. In particular, astrocyte-derived thrombospondins (TSPs) are large extracellular matrix proteins (450 kDa) that have been identified as major contributors to astrocyte-regulated excitatory synapse formation [17]. The TSP family consists of two subfamilies, A and B, according to their organization and domain structure [18, 19]; A includes the trimeric TSP-1 and TSP-2, while B includes the pentameric TSP-3, TSP-4 and TSP-5 [20, 21]. Recently, the *THBS1* gene, which encodes the TSP-1 protein, has been identified as an autism risk gene [22]. In the central nervous system (CNS), TSP-1 is mostly enriched in glia and predominantly expressed by developing astrocytes during early postnatal development in the rodent cortex [23], which correlates with the onset of synaptogenesis. TSP-1 regulates excitatory synaptogenesis through the gabapentin receptor *α*2δ-1 [24, 25], and neuroligin-1 in hippocampal neurons [26]. Double TSP-1 and −2 knockout mice show a reduced number of excitatory synapses in the cortex [17] and display dendritic spine irregularities [27]. Given that FMRP and TSP-1 are both expressed in immature astrocytes and have been associated with the timely development of synapses, there is reason to believe that the early regulation of synaptogenesis could be influenced by TSP-1.

Previously, our group has demonstrated that astrocytes modulate neuronal development in FXS; however, it is unknown whether the effects can be attributed to direct physical astrocyte-neuron interactions and/or to the release of extrinsic synaptic cues derived from astrocytes that are responsible for guiding development. Here, we determined the role of astrocyte-secreted TSP-1 on neuronal maturation and synaptic development in FXS. To explore the consequences of altered astrocyte signaling during development, we optimized an indirect (non-contact) astrocyte-neuron co-culture method with either astrocyte-conditioned medium or an astrocyte feeder layer to promote neuronal attachment and survival. Using this experimental paradigm, our results showed abnormal spine maturation and synapse development in normal hippocampal neurons grown with conditioned media and a feeder layer from FXS astrocytes. We also found that TSP-1 levels were markedly reduced in FXS cultured astrocytes and conditioned media. Further, the addition of TSP-1 to FXS cultures prevented synaptic and spine alterations. These findings provide insight into the significance of astrocyte-derived cues during early developmental periods in the brain that underlie the proper establishment of neural circuitry.

## Methods

### Animals

The FMRP mouse colony was established from breeding pairs of FVB.129P2(B6)-Fmr1^tm1Cgr^ mice. The wildtype (WT) and *Fmr1* knockout (KO) mice were maintained as individual strains and genotyped regularly. Both male and female mice were used in the experiments. The mice used for these experiments were housed and bred at the McMaster University Central Animal Facility. All experiments complied with the guidelines set out by the Canadian Council on Animal Care and were approved by the McMaster Animal Research Ethics Board.

### Hippocampal neuron isolation

Hippocampal neurons were obtained from embryonic day E15–17 (day of sperm plug counted as E1) WT and *Fmr1* KO animals. Hippocampal tissue was isolated from at least six embryonic pups, digested with 2.5 % trypsin, and triturated through a fire-polished glass Pasteur pipette. The neurons were subsequently plated on poly-_L_-lysine (1 mg/ml, Sigma) and laminin (0.1 mg/ml, Invitrogen) coated glass coverslips in 24-multiwell plates immediately after dissociation at a density of 20 000 cells per well in Neural Growth Medium consisting of Neurobasal (NB) (Invitrogen) enriched with 0.5 mM GlutaMAX (Invitrogen), and 2 % B-27 Supplement (Invitrogen). Neurons remained in culture for 17 days in vitro (*DIV*) to allow for the development and maturation of dendritic spines. Immunocytochemical studies subsequently ensued to identify alterations in spine density, morphology and length, and the formation of excitatory synapses.

### Primary cortical astrocyte cultures

Cortical astrocytes were prepared from four WT or *Fmr1* KO postnatal day 0 to day 2 (P0–P2) pups, as detailed previously by our laboratory [28]. Briefly, whole brains were extracted and cortical tissue was dissected and incubated with 2.5 % trypsin (Invitrogen) and 15 mg/mL DNase (Roche) at 37 °C. Following successive mechanical trituration using a serological pipette, the cells were passed through a 70 μm cell strainer (Fisher Scientific), dissociated into a single-cell suspension, and re-suspended in 10 % Glial Media (GM) comprised of Minimum Essential Medium (MEM) (Invitrogen), 0.6 % glucose and 10 % horse serum (Invitrogen). The astrocytes were seeded in a T75 flask and maintained in culture for 7–12 days in a humidified 5 % CO_2_, 95 % O_2_ incubator at 37 °C. Partial medium changes were performed every 2–3 days. Cultures consisted of at least 95 % astrocytes as determined by glial fibrillary acidic protein (GFAP) immunocytochemistry.

### Preparation of astrocyte-conditioned medium (ACM)

After 4–5 days in culture and achieving 50–65 % confluency, the monolayers of astrocytes were subjected to a Neuronal Growth Media (NGM) switch. The cultures were washed extensively with Neurobasal (Invitrogen) and the media was replaced with NB/B-27 culture medium to generate the ACM. In preparation for conditioning, ACM was harvested for 4 days and collected. The media was filtered using a 0.22 μm syringe filter for the removal of cellular debris and concentrated ten times by filtration through a 10 kDa molecular weight cut-off (MWCO) centrifuge concentrator (Satorius Stedim). ACM was stored at −80 °C and used at a final concentration of 5×. Notably, this procedure closely follows that of Christopherson et al. [17] used for the isolation of thrombospondins. The ACM was spun at 5000 rpm for 5 min to remove cell debris prior to protein quantification or plating with the neurons.

### Astrocyte feeder layer

To evaluate astrocyte non-contact mediated influences on morphological parameters, ThinCert™ cell culture inserts from Greiner Bio-One were utilized and placed in 24-multiwell plates to facilitate the growth of the cortical astrocytes. These culture inserts consist of a 0.4 μm porous, PET membrane support that forms a two-compartment system. In this set-up, cultures were co-cultivated with neurons and astrocytes in physically separated compartments to facilitate paracrine-signaling interactions involving diffusible astrocyte-secreted factors. For preparation of the astrocyte feeder layers (AFL), confluent astrocyte monolayer cultures were trypsinized with 0.05 % Trypsin-EDTA to lift adherent cells and subsequently passaged. The dissociated cells (9000 cells) were seeded into the cell culture inserts (positioned above the bottom of the well) and maintained in 10 % GM for 3 days. Media was switched to Neural Growth Media (NGM) at least one day before plating neurons. Subsequently, hippocampal neurons supplemented with NGM were plated below the cell culture inserts on the coverslips. NGM was replenished every 3–4 days.

### DiI neuronal labeling

Dendritic spines were identified using the well-characterized fluorescent marker DiI (1,1′-dioctadecyl-3,3,3′,3′-tetramethylindocarbocyanine perchlorate), as described by Cheng et al. [29]. In brief, the neurons were fixed with 2 % paraformaldehyde and stained with lipophilic DiI (Invitrogen). For the staining, 2–3 DiI crystals were applied to each coverslip. A small amount of Dulbecco’s Phosphate Buffered Saline (DPBS, Invitrogen) was dispensed to the edge of the wells to prevent dehydration of the cells. The neurons were stained for 10 min on an orbital shaker and copiously washed with DPBS to remove all crystals. The cells were incubated in DPBS overnight at room temperature. The following day, the coverslips were rinsed with dH_2_O, air-dried and mounted to slides with Prolong Gold Anti-fade (with DAPI) fluorescent mounting medium (Invitrogen). The cells were visualized 72 h from the initial staining to allow for the complete migration of the dye into the dendritic spines.

### Confocal imaging

Visual imaging of dendritic spines was acquired using a Zeiss 510 confocal laser-scanning microscope (LSM 510). All images were taken using a 63×/1.2 water immersion lens. A 543 nm Hene-1 Rhodamine laser was utilized to visualize the fluorescence emitted by DiI with the filter channel 3 BP560–615 nm. To view the specimen with reflected fluorescent light, the reflector turret was programmed to position F set 15 in correlation to the Rhodamine laser, and the single-track configuration was chosen. We used 1024 × 1024 pixels for image size and set the scan speed at a setting of 6. Scan direction and line averaging were also adjusted to a setting of 6. The pinhole diameter was configured to 1 Airy unit (124 μm). Series stacks were collected from the bottom to the top covering all dendrites and protrusions, with an optical slice thickness of 0.5 to 1 μm. The resulting images (4–6) were then reconstructed according to z-stack projections of the maximum intensity.

### Dendritic spine analysis

ImageJ software (RSB, NIH) was used for viewing the confocal images and for spine quantification. In order to increase the magnification for a better view of the spines without loss of image quality, the resolution of the stack image was increased by a factor of 5 in the X and Y directions with the plug-in Transform J Scale [30]. The length of a spine was obtained by drawing a line from its emerging point on the dendrite to the tip of its head. Approximately, 25 neurons were selected at random per condition across three coverslips from two batches of cultures per group were used for quantitative analysis. Secondary dendritic branches were selected for analysis. Density, morphology and length of spines were scored in dendritic segments 10 μm in length. Morphologically, two populations of spines were identified: (1) ‘mature’ stubby/mushroom-like spines, which are small, rounded spines with or without a short neck and (2) ‘immature’ filopodium-like spines, which are typically longer, thin and lack a head. Two to three independent experiments were performed. For each independent culture, 40–50 dendritic segments (10 μm in length) were selected and evaluated for spine analysis.

### Immunocytochemistry

Following 17 *DIV*, hippocampal neurons were fixed with ice-cold (−20 °C) acetone for 20 min and processed for immunocytochemistry. Cells were washed three times in PBS and permeabilized with 0.1 % Triton X-100, respectively. Non-specific binding was blocked with 1 % bovine serum albumin (BSA) with the appropriate normal animal serum for 30 min at room temperature (RT). Primary antibodies were applied to the coverslips and incubated overnight at 4 °C. The second day, following washes with PBS, secondary antibodies were incubated with the cells for 3 h at RT. Lastly, the coverslips were mounted on to slides with Prolong Gold Anti-fade fluorescent mounting medium (Invitrogen) containing DAPI to stain for nuclei. The following antibodies, diluted in PBS, were used: monoclonal synaptophysin (clone SVP-38; 1:250; Sigma-Aldrich), mouse monoclonal postsynaptic density 95 (PSD-95) (clone 6G6-1C9; 1:200; Millipore Bioscience Research Reagents), donkey anti-mouse AlexaFluor 594 (1:1500, Life Technologies), and goat anti-rabbit FITC (1:100, Jackson ImmunoResearch).

Primary cortical astrocytes were also processed in a similar manner to visualize the localization of TSP-1 at 7 *DIV*. The following antibodies were used to label the astrocytes: mouse monoclonal thrombospondin-1 (A6.1; 1:100; ThermoFisher Scientific), polyclonal rabbit anti-GFAP (1:500, Dako), donkey anti-mouse AlexaFluor 594 (1:1500, Life Technologies), and goat anti-rabbit FITC (1:100, Jackson ImmunoResearch).

### Synaptic puncta analysis

Images were acquired using a Zeiss AxioImager.M2 at 40× magnification configured with the ApoTome.2 and Zeiss Zen Blue image acquisition software. Synapses were identified by the co-localization of the pre-(synaptophysin) and post-synaptic (PSD-95) puncta, using a custom written plug-in for ImageJ. After selecting a 50 μm segment along the dendrite, the image was processed using the above plug-in. Briefly, low frequency background from each channel (red and green) of the image was removed with the rolling ball background subtraction algorithm. Then, the puncta in each single-channel was ‘masked’ by thresholding the image so that only puncta remained above threshold. Puncta were then identified in each channel by the “Puncta Analyzer” plug-in for Image J. Co-localization of the puncta in each channel was identified when the distance between the centers of the two puncta was less than the radius of the larger puncta. The number of co-localized puncta was then recorded as synapses. Two to three independent experiments were performed. Approximately 20–25 neurons from at least two coverslips were analyzed per condition/group. For each independent culture, 45–50 (50 μm long) segments along the dendrites were evaluated for synaptic protein co-localization. Multiple dendritic segments were sampled within a given neuron. The PSD95/synaptophysin-immunoreactive puncta along the dendrites in each photomicrograph were counted manually and normalized by dendrite length. The photography and analysis of immunoreactivity were conducted in an investigator-blinded manner.

### Measurements of TSP-1 expression and release

TSP-1 levels were determined in astrocyte cultures and astrocyte-conditioned medium (ACM). Confluent astrocyte cultures were incubated with 0.05 % Trypsin-EDTA for 5 min at 37 °C. Once cells were fully lifted, media with serum was added to stop the digestion. The cells were centrifuged at 250 g for 5 min, washed with ice-cold PBS, and lysed with cell lysis buffer containing 50 mM Tris–HCl, 150 mM NaCl, 1 % Triton-X and 5 mM EDTA with protease inhibitors (Roche). The lysates were collected in Eppendorf tubes, centrifuged at 12000 rpm in an Eppendorf microcentrifuge for 20 min at 4 °C, and the supernatants were used to determine the intracellular concentration of TSP-1. Aliquots of ACM from the same cultures were also processed to determine secreted, extracellular levels of TSP-1. TSP-1 protein measurements were determined using the Mouse Thrombospondin-1 ELISA kit (Biotang, Inc) following the vendor’s instructions. Sample protein content normalization was based on total protein concentration, which was determined for each sample by Bradford assay.

### Thrombospondin-1 treatments

To assess the neuromodulatory effect of exogenous TSP-1 on spine development, hippocampal neuronal cultures derived from *Fmr1* KO mice were treated with human recombinant TSP-1 (R&D System) to stimulate spine formation. Recombinant TSP-1 was added to the neuronal media one day after plating, and replenished every 3 days for 17 days. A dose concentration of 250 ng/ml was used to restore TSP-1 levels [27]. Control cultures were incubated with heat-inactivated TSP-1 (TSP1-HI, 100 °C for 5 min), or gabapentin (GBP; 32 μM) [24] to inhibit astrocyte induced spine and synapse formation.

### Statistics

Statistics were performed using GraphPad Prism 5.0. Differences were detected using a Student’s *t*-test or one-way analysis of variance (ANOVA). Following one-way ANOVA, post hoc differences were resolved using the Bonferroni correction. A *p*-value of <0.05 was considered significant. All values are expressed as mean ± SEM; *n* = number of neurons (unless otherwise specified); *N* = number of experiments.

## Results

### *Fmr1* knockout neurons display dendritic spine and synaptic abnormalities relative to their wildtype counterparts

**Fig. 1:**
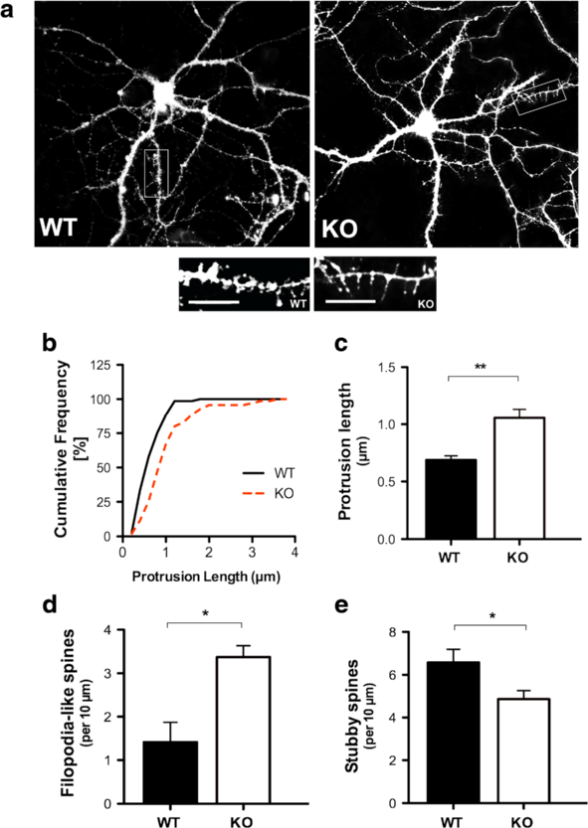
Spine length is altered in *Fmr1* KO hippocampal neurons at 17 *DIV*. **a** Representative images of DiI labeled WT (left panel) and *Fmr1* KO (right panel) neurons highlighting dendrites and spines. Inset is an example of a 50 μm segment used for morphological analysis and quantification. Note the high prevalence of filopodium-like spines in the *Fmr1* KO dendritic segment (right panel) relative to the WT segment (left panel). **b** Cumulative frequency distribution of spine lengths comparing *Fmr1* KO and WT neurons. **c** *Fmr1* KO neurons display increased dendritic spine length compared to their WT counterparts. **d** Assessment of spine morphology in *Fmr1* KO neurons shows a significant increase in the number of filopodia-like spines and **e** a reduction in the number of stubby spines. Data was analyzed by Student’s *t*-test. Data are presented as the mean ± SEM, *p < 0.05, **p < 0.01; *n* = 75 neurons per group, *N* = 3 independent experiments. Scale bars: 10 μm

Previous reports have established the critical role of astrocytes in FXS [9, 10]; however, the role of astrocytes in regulating dendritic spine formation has not been elucidated. To investigate morphological differences, we first examined dendritic spine subtypes and length in dissociated hippocampal neuronal cultures labeled with DiI from *Fmr1* knockout (KO) and wildtype (WT) mice at 17 days in vitro (*DIV*). DiI is a fluorescent carbocyanine membrane dye that effectively illuminates the cellular architecture of neurons and individual processes, including dendrites and spines [31]. The morphological analysis of the neurons was conducted at 17 *DIV* to provide sufficient time for spine development and maturation, and circumvent the loss of neuronal processes due to the instability of long-term cultures. For simplicity, we categorized both the stubby and mushroom-shaped (mature) spines as “stubby”, and thin and filopodium spines as ‘filopodia-like’ (immature) spines. Dendritic spine length of each individual dendritic spine was measured from its point of insertion in the dendritic shaft to the distal tip of the spine, while rotating the image in 3D. Figure 1a shows representative images of neurons and dendritic segments from *Fmr1* KO and WT mice.

Consistent with findings from the literature, we found a clear difference in dendritic spine length between WT and *Fmr1* KO hippocampal neurons. *Fmr1* KO neurons displayed remarkably longer spines (~1.5 fold increase) relative to their WT counterparts at 17 *DIV* (Fig. 1b and c, *p* < 0.01). The structural profiles of the spines also differed considerably between the WT and *Fmr1* KO hippocampal neurons. *Fmr1* KO neurons displayed a higher density of immature thin/filopodia-like spines (Fig. 1d, *p* < 0.05), and a striking decrease in the number of mature stubby subtypes compared to WT hippocampal neurons (Fig. 1e, *p* < 0.05). The increased prevalence of elongated spines, characteristic of immature synapses, suggests that dendritic development is delayed in the *Fmr1* KO neurons. Notably, a slight decrease in the overall spine density was observed in the F*mr1* KO neurons; however, the differences were not statistically significant (*p* > 0.05, Additional file 1: Figure S1).

**Fig. 2:**
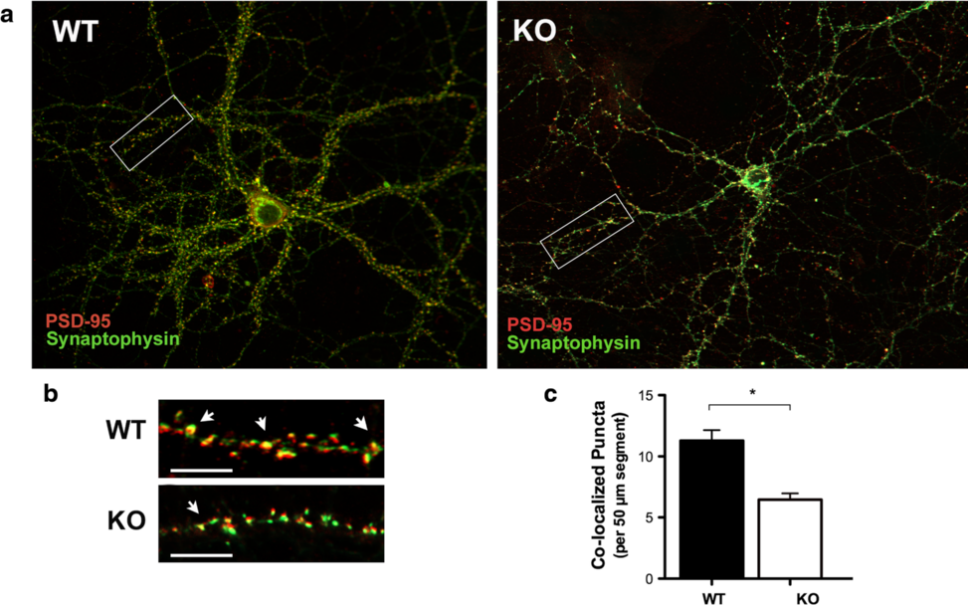
Quantification of excitatory synapse formation at 17 *DIV*. **a** Representative images of WT and *Fmr1* KO neurons visualized using immunofluorescence. Inset is an example of a 50 μm segment used for excitatory synapse quantification. **b** Excitatory synapses are denoted by the arrows representing the co-localization of pre-(synaptophysin, SYN, 1:250, green) and post-synaptic (PSD-95, 1:200, red) puncta (arrows). Synapses were identified using a custom written plug-in for ImageJ (https://imagej.nih.gov/ij/) – “Puncta Analyzer”. **c** At 17 *DIV*, *Fmr1* KO neurons exhibit a reduction in the number of co-localized puncta relative to their WT counterparts. Data was analyzed by Student’s *t*-test. Data are presented as the mean ± SEM, *p < 0.05; *n* = 75 neurons per group, *N* = 3 independent experiments

Next, we sought to examine the role of FMRP in regulating excitatory synapse formation. The establishment of neural circuitry requires vast numbers of synapses to be generated during a specific window of brain development. We used immunocytochemistry with antibodies directed at synaptophysin (a presynaptic protein that exists as part of the synaptic vesicle membrane) and PSD-95 (a postsynaptic protein that forms part of the postsynaptic density) to identify synaptic protein aggregates in neurons at 17 *DIV*. Synapses were detected using the “Puncta Analyzer” plug-in for ImageJ. The co-localization of immunoreactivity to pre- and postsynaptic protein markers is denoted by yellow puncta (spots of intense staining). Each yellow punctum corresponded to the structural site of a single functional synapse (Fig. 2a and b). At 17 *DIV*, the clustering of both pre- and postsynaptic proteins was decreased with less co-localization of puncta observed in *Fmr1* KO neurons relative to their WT counterparts (Fig. 2c, *p* < 0.05). These results suggest that excitatory synapse formation is altered in *Fmr1* KO hippocampal neurons.

### WT hippocampal neurons exhibit morphological spine and synaptic deficits and when grown with an *Fmr1* KO astrocyte feeder layer

**Fig. 3:**
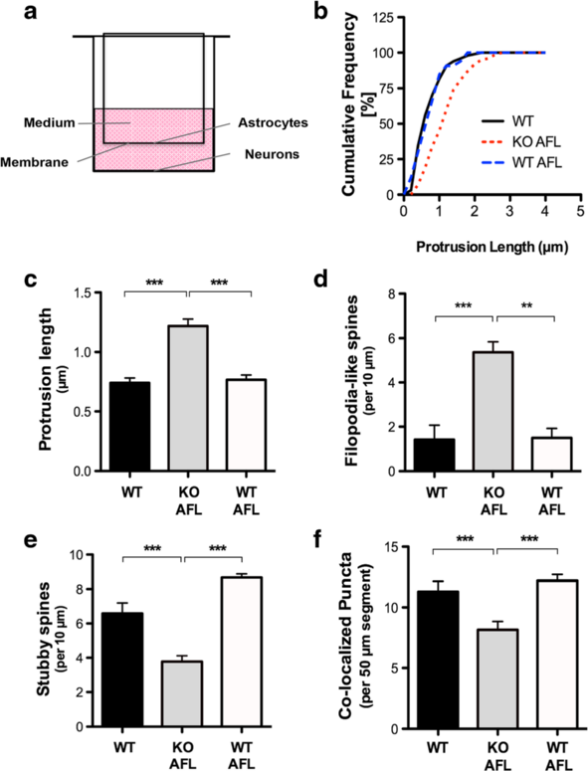
WT hippocampal neurons display morphological spine deficits when grown in the presence of an *Fmr1* KO astrocyte feeder layer. **a** Schematic illustrating the indirect astrocyte-neuron co-culture set-up. Astrocytes were grown in a cell culture insert with a permeable membrane facing the neurons and sharing the same, defined medium. **b** Cumulative frequency distribution of spine lengths comparing WT neurons grown independently and in co-culture with WT or *Fmr1* KO astrocyte feeder layers (AFL) at 17 *DIV*. **c** WT hippocampal neurons grown without direct contact with *Fmr1* KO astrocytes present with longer spines. **d** Assessment of spine morphology in *Fmr1* KO AFL/WT neuron co-cultures indicates a significant increase in the density of filopodia-like spines and (**e**) a decreased density of stubby spines. Spine density represents the average number of spines scored in a 10 μm dendritic segment. **f** *Fmr1* KO AFL/WT neuron co-cultures exhibit a reduction in the number of co-localized synaptic puncta relative to WT neurons cultured alone or with a WT AFL (*p* < 0.01). Excitatory synapses represent the average number of co-localized puncta scored in a 50 μm dendritic segment. One-way ANOVA with Bonferroni correction was used to analyze the data. Data are presented as the mean ± SEM, **p < 0.01, ***p < 0.001; *n* = 50 neurons per group, *N* = 2 independent experiments

Membrane-bound factors are known participants in synaptogenesis; however, the contributions of diffusible astrocyte factors on neuronal development in FXS have not been elucidated. To explore the possible role of diffusible factors, we plated hippocampal neurons on glass coverslips positioned below a feeder layer of astrocytes (AFL), facilitating the exposure of soluble factors to neurons through a membrane without direct contact between these two cell types (Fig. 3a). Using this non-contact co-culture approach, we found that deficits in FMRP in astrocytes affected the development of spines at 17 *DIV*. Although no significant effects were observed on spine density, we found that WT neurons exhibited morphological deficits when grown in the presence of an *Fmr1* KO feeder layer. WT hippocampal neurons cultured below an *Fmr1* KO feeder layer displayed abnormally longer spines (Fig. 3b, ~1.6 fold increase) than WT neurons grown independently (Fig. 3c, *p* < 0.001) or with a WT feeder layer (Fig. 3c, *p* < 0.001). In addition, WT neurons exhibited a striking increase in the density of immature filopodia-like spines (Fig. 3d, *p* < 0.001) when cultured in combination with an *Fmr1* KO feeder layer. WT hippocampal neurons grown in the presence of an *Fmr1* KO feeder layer also presented with a reduced proportion of stubby spines that are typically indicative of mature spines (Fig. 3e, *p* < 0.001). Notably, WT neurons supplemented with a feeder layer of normal astrocytes displayed the highest proportion of stubby spines, suggesting that WT astrocytes promote spine maturation. Conversely, the increased frequency of ‘immature’ thin spine phenotypes, reminiscent of immature spine precursors, filopodia, suggests that *Fmr1* KO astrocytes alter the appropriate formation of dendritic spines in WT neurons. Further, the observed spine phenotypes in the WT neurons cultured with the *Fmr1* KO feeder layer resemble the structural deficits observed in *Fmr1* KO neurons, demonstrating a role for secreted signals on spine development.

Next, we sought to ascertain the ability of ACM or astrocyte-feeding layers to induce the development of synapses in neurons (Fig. 3f). WT neurons grown in the presence of an *Fmr1* KO feeder layer displayed significantly reduced co-localization relative to WT neurons cultured alone (*p* < 0.05) or with a WT AFL (*p* < 0.001). These results demonstrate that synaptic protein clustering is impaired when WT neurons are grown with a feeder layer derived from *Fmr1* KO astrocytes.

### Spine and synaptic protein abnormalities are prevented in *Fmr1* KO neurons by WT astrocyte conditioned media or a WT feeder layer

**Fig. 4:**
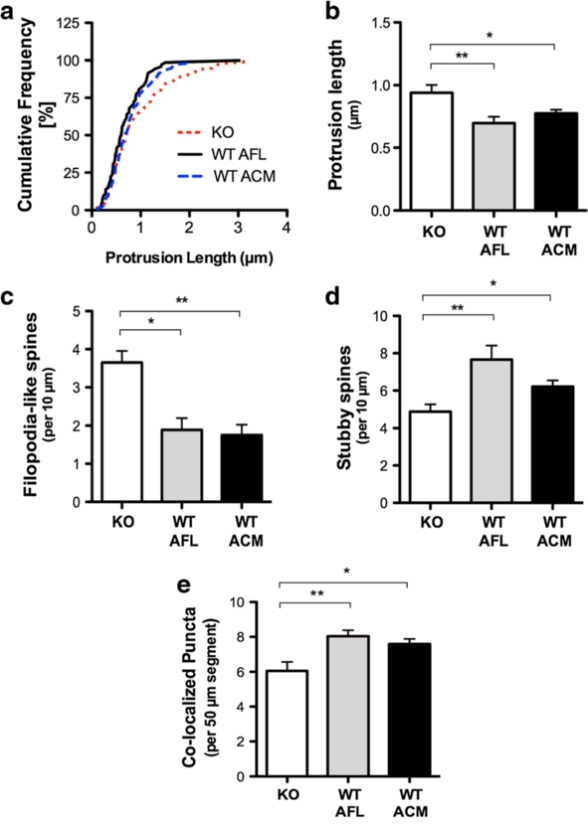
Morphological abnormalities in *Fmr1* KO hippocampal neurons are corrected by WT astrocytes with indirect co-culture. **a** Cumulative frequency distribution of spine lengths comparing *Fmr1* KO neurons grown in the presence of a WT AFL or supplemented with WT ACM at 17 *DIV*. **b** Spine length is significantly reduced in *Fmr1* KO neurons cultured with WT ACM or WT AFL. **c** Co-cultures with WT AFL or WT ACM reduced the proportion of immature spine phenotypes in the *Fmr1* KO neurons. **d** Alternatively, WT ACM and WT AFL induced an increase in the formation of mature spines. **e** *Fmr1* KO neurons supplemented with WT ACM increases the co-localization of excitatory pre- and postsynaptic proteins, synaptophysin and PSD-95. Similarly, WT AFL/*Fmr1* KO neuron co-cultures enhance the number of co-localized puncta. One-way ANOVA with Bonferroni correction was used to analyze the data. Data are presented as the mean ± SEM, *p < 0.05, **p < 0.01; *n* = 50 neurons, *N* = 2 independent experiments

Given that *Fmr1* KO astrocytes impact the dendrite morphology of cultured WT hippocampal neurons, we tested whether secreted molecules from WT astrocytes are able to prevent the abnormal spine development in *Fmr1* KO hippocampal neurons. We analyzed the structural morphology of dendritic spines in *Fmr1* KO neurons cultured with either conditioned media or a feeder layer derived from WT astrocytes. The application of ACM to cultures has been reported to be equally effective in inducing synapses as a feeder layer of astrocytes [32]. Synaptogenic factors are constitutively released from astrocytes grown in isolation in culture and do not require a neuronal signal to stimulate release [33, 34]. Our results showed that *Fmr1* KO neurons displayed an overall increase in spine length (Fig. 4a) compared to *Fmr1* KO neurons cultured with WT ACM (*p* < 0.05) or a WT feeder layer (*p* < 0.05). Remarkably, aberrations in spine length were mitigated in the *Fmr1* KO neurons when supplemented with either WT ACM by −1.2 fold (Fig. 4b, *p* < 0.05) or a WT feeder layer by −1.4 fold (Fig. 4b, *p* < 0.01). Furthermore, WT ACM significantly reduced the frequency of immature-appearing spine phenotypes in the *Fmr1* KO neurons (Fig. 4c, *p* < 0.01), restoring the density of unstable spines to normal levels. Similarly, a WT feeder layer considerably decreased the number of filopodia-like spines (*p* < 0.05). WT ACM also promoted spine maturation by inducing the formation of stubby/mushroom spines in the *Fmr1* KO neurons following 2.5 weeks in culture (Fig. 4d, *p* < 0.05). Likewise, *Fmr1* KO neurons grown with a WT feeder layer significantly increased the density of stubby spines (*p* < 0.01). Together, these results suggest that astrocyte-mediated signaling is an important regulator of spine development and is sufficient to rescue deficits in neuronal connections associated with FXS.

Next, given that soluble factors derived from FMRP-deficient astrocytes affect excitatory synapse formation, we investigated whether normal astrocytes could prevent the alterations observed in *Fmr1* KO neurons. Consistent with previous studies showing that ACM induces synapse formation [17], we found that the addition of WT ACM to *Fmr1* KO neurons significantly increased the number of synaptophysin/PSD95 co-localized puncta per 50 μm length of dendrite (Fig. 4e, *p* < 0.05). Our results also showed that a greater number of synaptic contacts were formed in *Fmr1* KO neurons supplemented with a WT feeder layer (*p* < 0.01). Together, these results demonstrate that synaptic protein clustering in *Fmr1* KO neurons can be restored to normal levels in the presence of FMRP-expressing astrocytes and further suggest a role for secreted factors in regulating synaptic development.

### Intracellular and extracellular TSP-1 expression levels are decreased in *Fmr1* KO astrocytes

**Fig. 5:**
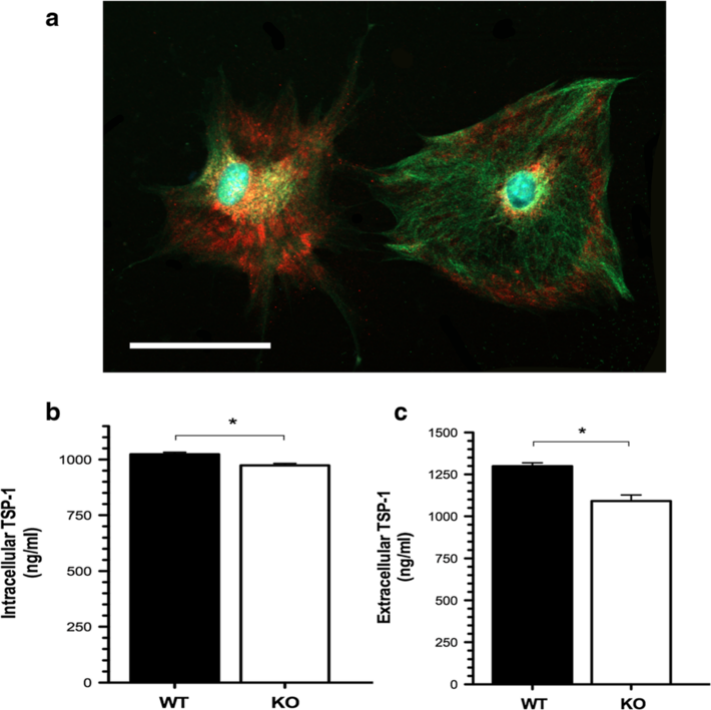
Reduced TSP-1 levels in cultured FXS cortical astrocytes. **a** Representative staining of cultured cortical astrocytes co-labeled with GFAP and TSP-1. At 7 *DIV*, the cells were fluorescently labeled with anti-GFAP (glial fibrillary acidic protein, green) to visualize astrocytes and anti-TSP-1 (thrombospondin-1, red) to identify the intracellular expression of TSP-1 using a 20× objective. Nuclei were counterstained with DAPI (blue). Cultured cortical astrocytes express abundant levels of TSP-1 at 7 *DIV*. Scale bars: 50 μm. **b** Quantification of intracellular TSP-1 protein levels by ELISA in cellular lysates. *Fmr1* KO astrocytes express lower levels of cellular TSP-1 compared to their WT counterparts. **c** Quantification of extracellular secreted TSP-1 in astrocyte-conditioned media. Measurements in TSP-1 protein levels in ACM revealed marked reductions in secreted TSP-1 by *Fmr1* KO astrocytes. Data was analyzed by Student’s *t*-test. Data are presented as the mean ± SEM, *p < 0.05; *n* = 5 cultures per genotype

TSP-1 is a matricellular protein synthesized and released by astrocytes during early development of the nervous system [35, 36]. TSP-1 promotes neurite outgrowth and survival [37–39], neuronal migration [40] and synaptogenesis [17] in vivo and in vitro. It participates in synaptic remodeling following injury [41] and is required for synaptic and motor recovery after stroke [42], suggesting that TSP-1 also participates in neuronal plasticity. Since TSP-1 has been identified as a major contributor to astrocyte-regulated excitatory synapse formation [17, 43], we determined if the expression of TSP-1 was altered in FXS cortical astrocytes. Here, we found relatively high levels of TSP-1 expression in cortical astrocytes (Fig. 5a). However, quantification of TSP-1 protein levels by ELISA showed marked reductions in both cellular lysates and conditioned media derived from *Fmr1* KO astrocytes. In fact, *Fmr1* KO astrocytes express 5 % less cellular TSP-1 compared to their WT counterparts (Fig. 5b: 49.98 ± 13.24 ng/ml; *p* < 0.05); and secrete 20 % less TSP-1 into the media relative to their WT counterparts (Fig. 5c: 208.12 ± 40.77 ng/ml; *p* < 0.05). These results demonstrate that TSP-1 expression and release are both altered in *Fmr1* KO astrocytes, suggesting that a lack of FMRP expression prevents normal TSP-1 expression in cortical astrocytes during development.

### Addition of TSP-1 corrects abnormal dendritic spine development and excitatory synapse formation in *Fmr1* KO neurons

**Fig. 6:**
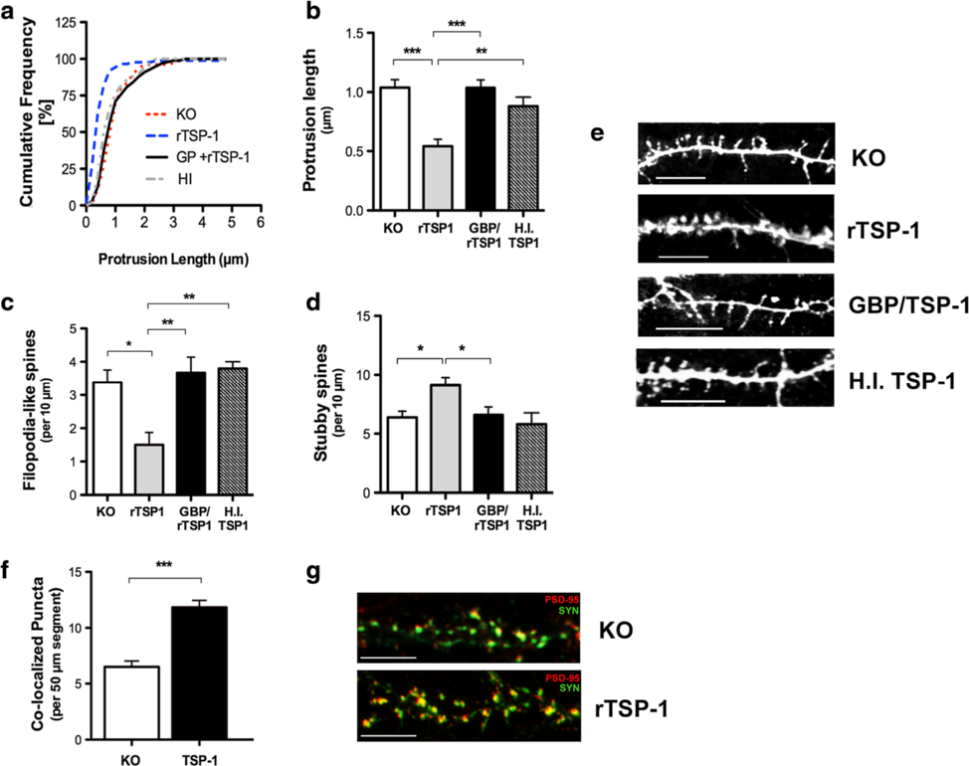
TSP-1 prevents spine alterations in *Fmr1* KO neurons. Analysis of the effects of TSP-1 administration on dendritic spine morphology in *Fmr1* KO hippocampal neurons at 17 *DIV*. Continuous treatment with recombinant TSP-1 (250 ng/ml) averted the spine alterations in *Fmr1* KO neuronal cultures. **a** Cumulative frequency distribution of spine lengths. **b** rTSP-1 induced a significant reduction in spine length and (**c**) the density of filopodia-like spines in the *Fmr1* KO neurons. **d** A marked increase in the density of stubby spines was observed following the application of rTSP-1. Heat-inactivated human recombinant TSP-1 (HI-TSP1) or gabapentin (GBP; 32 ug) in combination with rTSP-1 did not exert a neuroprotective effect on spines. One-way ANOVA with Bonferroni correction was used to analyze the data. All data are expressed as mean ± SEM. *p < 0.05; *n* = 75 neurons per group, *N* = 3 independent experiments. **e** Dendritic segments labeled with fluorescent DiI to illustrate typical spine morphologies found in *Fmr1* KO neurons and following treatment with exogenous TSP-1, GBP/TSP-1 or HI-TSP-1, respectively. Scale bars: 10 μm. **f** Excitatory synapse formation is normalized in *Fmr1* KO following rTSP-1 treatment. Data was analyzed by Student’s *t*-test. Data are presented as the mean ± SEM, ***p < 0.001; *n* = 75 neurons per group, *N* = 3 independent experiments. **g** Double immunofluorescence of dendritic segments with PSD-95 (red) and synaptophysin (green) to identify excitatory synapses. Scale bars: 10 μm

Given the disruption in TSP-1 expression in *Fmr1* KO astrocytes and the aberrant growth of neurons in the presence of these cells, we examined the effects of exogenous TSP-1 on dendritic spine morphology in cultured hippocampal neurons. *Fmr1* KO hippocampal neurons were treated with human recombinant (rTSP-1; dosage of 250 ng/ml) to stimulate spine formation [27]. Treatment with TSP-1 was well tolerated by the hippocampal neurons, with no neurotoxicity at concentrations as high as 1000 ng/ml. Interestingly, the addition of rTSP-1 to *Fmr1* KO neurons mimicked the restorative effects of a WT feeder layer or WT ACM. Prior to rTSP-1 treatment, an increased frequency of long, thin spines were observed in *Fmr*1 KO neurons (Fig. 6a and b, *p* < 0.01). Following TSP-1 treatment, spine protrusion length was significantly reduced by 50 % in *Fmr1* KO neurons (Fig. 6b, *p* < 0.001). Treatment with rTSP-1 decreased the proportion of thin spines (Fig. 6c, *p* < 0.05) and promoted the formation of mature spines in 2.5 week-old cultures (Fig. 6d, *p* < 0.05). To test whether the reduction in astrocytic TSP-1 was indeed responsible for the aberrant spine morphology, cultured neurons were treated with either heat inactivated TSP-1 (TSP1-HI) or rTSP-1 co-incubated with gabapentin (GBP), a competitive TSP-1 receptor antagonist. Notably, morphological spine anomalies were not corrected in *Fmr1* KO neurons treated with TSP1-HI or rTSP-1 (250 ng/ml) with gabapentin (GBP; 32 ug), further confirming the specific role of active TSP-1 on *Fmr1* KO hippocampal neurons.

To elucidate the neuromodulary effects of TSP-1 on excitatory synapse formation, *Fmr1* KO neurons were treated with a dose of 250 ng/ml of rTSP-1. At 17 *DIV*, an increase in the co-localization of pre-and post-synaptic puncta was observed when *Fmr1* KO neurons were treated with rTSP-1 (Fig. 6f and g; *p* < 0.001). Therefore, these results support the concept that rTSP-1 can increase the number of both presynaptic and postsynaptic protein clusters, contributing to the recovery of the alterations in excitatory synapses in *Fmr1* KO neurons.

## Discussion

Dendritic spine abnormalities have long been recognized as structural correlates of learning impairments in FXS and various forms of ASDs [44, 45]. However, the origin and mechanisms involved in spine dysgenesis are not well understood. In contrast to the expression of FMRP in neurons, relatively little is known about the function of FMRP in glial cells during CNS development. In these particular experiments, we investigated the role of astrocytes in the development of the abnormal dendritic spine morphology and synaptic aberrations seen in FXS. Primary hippocampal neurons were grown in a non-contact co-culture set-up with astrocytes for 17 *DIV*, allowing for the continuous exchange of molecules between both cell types via the shared defined medium. In this setting, we focused on the contribution of secreted CNS matricellular molecules to neuronal development and synapse formation using an *Fmr1* knockout (KO) Fragile X mouse model. Here, we show that: i) *Fmr1* KO astrocytes display deficits in TSP-1 levels; ii) An altered expression of astrocyte-secreted factors plays a significant role in dendritic spine dysgenesis and excitatory synapse formation; iii) Exogenous TSP-1 reverts *Fmr1* KO astrocyte-mediated spine and synaptic alterations to normal levels.

### Role of astrocytes in fragile X neurobiology

Astrocytes are important regulators of neuronal growth and maturation, and are key players in the abnormal neurobiology of a number of developmental disorders, including FXS [27, 46, 47]. In cultured hippocampal neurons, astrocytes have been shown to be a critical component for appropriate dendritic spine morphology and the ability of neurons to form synapses [48–51]. Several studies have recently linked the absence of FMRP in astrocytes to abnormal neuronal structure and function in FXS. Our previous work showed that the loss of FMRP in astrocytes causes abnormal dendritic morphology, including aberrant dendritic branching and dysfunctional synapse development [9, 10]. It has also been shown that the selective loss of FMRP from astrocytes (but not neurons) leads to the reduced expression of the glutamate transporter GLT-1 (EAAT2) and subsequent reduced glutamate uptake by these cells [52]. In this study, we confirmed that synapse formation between neurons is driven by factors released from astrocytes, and astrocytes devoid of FMRP affect proper spine development. More importantly, factors derived from normal astrocytes protect the loss of synapse formation, decrease the density of immature spines, and promote mature spine formation in *Fmr1* KO neurons.

Under normal conditions, the release of soluble factors from astrocytes guide neurite growth, synapse formation and maturation [34, 53]. In FXS, dendritic contacts fail to mature and the result is aberrant neural connectivity. We hypothesized this was due to a deficit in astrocyte-derived factors since *Fmr1* KO astrocytes grown with WT neurons lacking direct contact contributed to the abnormal spine morphology, evidenced by a greater number of longer, thinner spines. In support of this, a recent study by Yang et al. [54] showed that elevated levels of neurotrophin-3 in *Fmr1 KO* astrocytes partially altered dendritic growth in neurons. Therefore, we aimed to identify potential additional astrocyte-derived soluble factors that contribute to the spine development and maturation impairment in FXS. Our study revealed that *Fmr1* KO astrocytes show a notable deficit in the expression of soluble factor TSP-1, resulting in dendritic spine dysgenesis in *Fmr1* KO neuronal cultures that can be corrected with the exogenous addition of TSP-1.

### Thromobspondin-1 contributes to astrocyte-regulated synapse formation

TSPs have been previously identified as important CNS synaptogenic molecules. TSP-1 and −2 are transynaptic organizers, highly expressed by immature astrocytes, which strongly promote structural synapse formation [55]. In particular, they act as permissive switches that time CNS synaptogenesis and enable neuronal molecules to assemble into synapses within a specific window of CNS development. This narrow developmental window typically coincides with the initiation of nascent synaptic contacts between dendrites and axons. Therefore, TSP-1 and −2 serve a transient function and are not required for maintenance of synapses in the adult. Both in vitro and in vivo studies have demonstrated the capacity of TSPs to increase synapse number, promote the localization of synaptic molecules, and refine the pre- and postsynaptic alignment [17, 56]. Furthermore, TSP1/2-deficient mice display a significant decrease in the frequency of excitatory synapses [17]. TSPs have also been shown to regulate presynaptic function by limiting presynaptic release at glutamatergic synapses, serving as a protective mechanism against excitotoxicity [25]. Recently, two transmembrane molecules were uncovered in mediating TSP-induced synaptogenesis. Firstly, neuroligin 1 interacts with TSP-1 and mediates the acceleration of synaptogenesis in young hippocampal neurons [26]. Secondly, α2δ-1, a subunit of the L-type Ca^2+^ channel complex (LTCC), was also identified as the postsynaptic receptor of TSP in excitatory CNS neurons [24]. Interaction between TSP and α2δ–1 sequentially recruits synaptic scaffolding molecules and initiates synapse formation [57]. Several other known receptors for TSP include the CD47 integrin- associated protein (CD47/IAP), a variety of integrins, and the low-density lipoprotein receptor-related protein LRP1; however, the role of each of these interacting proteins have not been elucidated.

### Deficits in TSP-1 affect synapse and dendritic spine development

Our findings revealed a decrease in TSP-1 protein expression in cellular lysates and in astrocyte-conditioned media from *Fmr1* KO astrocytes, which suggests that the lack of TSP-1 derived from FMRP-deficient astrocytes interferes with proper spine development and synapse formation in FXS. Likewise, deficits in TSP-1 expression in astrocytes has also been implicated in Down Syndrome (DS) from human brain tissue samples and DS cultured astrocytes [27]. In a study by Jayakumar et al. [58], a shortage of astroglial-derived TSP-1 contributed to synaptic dysfunction in hepatic encephalopathy, which was linked to a decrease in the neuronal expression of synaptophysin, synaptostagmin, and PSD-95, all of which are critically involved in maintaining the integrity of glutamatergic synapses. In line with these findings, we observed that a decrease in TSP-1 expression resulted in the reduced co-localization of pre- and post-synaptic synaptophysin and PSD-95 in *Fmr1* KO neurons.

Spine morphology is linked to synapse function and mushroom-shaped spines are considered to represent the most mature and stable spine morphology [59, 60]. Several categories of spines have been identified based on their shape and size, including thin, stubby, cup, and mushroom-shaped. Recent studies suggest that excitatory synapses are a component of mushroom-shaped dendritic spines [61]. Thus, the increase in mushroom-shaped spines in response to TSP-1 treatment is consistent with our findings that TSP-1 stimulates the formation of excitatory synapses. In our study, we also found that spine length, a measure of synaptic immaturity, was increased in *Fmr1* KO mice, suggesting a delay in the development of spines. The prevalence of thin, elongated dendritic spine morphology in *Fmr1* KO mice is reminiscent of that observed during early synaptogenesis [62] in the developing brain, as well as the morphology seen following sensory deprivation [63, 64]. The increase in immature-appearing spines could be due to augmented spine turnover [64], which fails to decrease in the early postnatal weeks in FXS [5]. Alternatively, the abundance of immature-looking spines in the *Fmr1* KO mice could be caused by a failure of a subset of spines to mature.

Interestingly, significant differences in overall spine density between the WT and *Fmr1* KO neurons were not observed. Similar studies in *Fmr1* KO mice have also found normal hippocampal spine densities [65] and individual spines with longer lengths [66]. In contrast, others have reported decreased spine density in the *Fmr1* KO mice compared to WT littermates [67, 68] and spines of normal length [67]. However, the discrepancies are likely attributed to the use of different staining and quantification methods of spines, and the choice of tissue source (cultured hippocampal neurons versus in vivo or ex vivo brain tissue). As we observed for hippocampal neurons, Braun et al. [62] also reported a lower abundance of excitatory synapses in *Fmr1* KO hippocampal neurons relative to WT at 2 weeks in culture. The failure of excitatory synapse maturation has also been noted in cortical neurons of *Fmr1* KO mice [69–71], as well as in *Fmr1* KO cerebellar granule cells [72].

Severe learning deficits have been associated with synaptic dysfunction in FXS and intellectual disabilities [73–75]. Here, we showed that deficits in the formation of dendritic spines in *Fmr1* KO neurons could be normalized with conditioned media or a feeder layer derived from normal astrocytes. Similarly, we demonstrated that the addition of exogenous TSP-1 to neuronal cultures markedly promoted the formation of mature (mushroom-shaped) dendritic spines and restored the number of synaptic protein clusters in *Fmr1* KO neurons comparable to the level that of WT neurons at 17 *DIV*. Notably, the application of ACM + TSP-1 has been shown to increase synaptic puncta to the same extent as either ACM or TSP-1 alone, indicating that TSP-1 alone is sufficient to recover synapse formation [17]. Mechanistically, TSP-1 may exert its restoring role in *Fmr1* KO neurons by specifically counteracting the loss in FMRP on the dendritic spines that are immature as a result of FMRP loss. Therefore, these findings identify TSP-1 as a critical astrocyte-secreted factor that modulates spine morphology and provide evidence that astrocytes are important contributors to synaptogenesis within the developing CNS. As such, defects in the secretion of astrocyte-derived TSP-1 during a crucial window of development likely contribute to the abnormal neurobiology seen in FXS. The potential therapeutic role of TSP molecules in excitatory synaptogenesis and their relevance to learning and memory in FXS will be an exciting avenue of future investigation.

Correspondingly, these findings highlight the important role of FMRP in astrocytes during the early postnatal weeks of synaptic development in FXS. However, the full extent of the cellular and molecular interactions that govern non-contact-mediated synaptogenic signaling between astrocytes and neurons is not clear. For instance, WT neurons cultured with an astrocyte feeder layer derived from *Fmr1* KO mice exhibit fewer mature spines than WT neurons cultured alone. These results suggest that *Fmr1* KO astrocytes may potentially release inhibitory substances that prevent normal spine development. Given that profound changes in both excitatory and inhibitory neurotransmission have been reported in FXS [76], the mechanisms involving a lack of factor and/or the exertion of inhibitory effects warrant further investigation. Therefore, studies exploring the contributions of additional factors may provide greater insight into the underlying mechanisms of FXS.

## Abbreviations

ACM, astrocyte-conditioned media; AFL, astrocyte feeder layer; CNS, central nervous system; *DIV*, days in vitro; *Fmr1*, *fragile x mental retardation gene 1*; FMRP, fragile x mental retardation protein; FXS, fragile X syndrome; GBP, gabapentin; HI, heat-inactivated; KO, knockout; PSD-95, postsynaptic density 95; rTSP-1, recombinant thrombospondin-1; SYN, synaptophysin; TSP-1, thrombospondin-1; WT, wildtype

## Additional file

Additional file 1: Figure S1. Quantification of spine density between WT and *Fmr1* KO hippocampal neurons. *Fmr1* KO neurons display a decrease in spine density compared to WT neurons; although no significant statistical differences were detected (*p* > 0.05). (TIFF 3706 kb)
