# Supplementary figures and images for: Astrocyte-secreted thrombospondin-1 modulates synapse and spine defects in the fragile X mouse model
Source: Mol Brain. 2016 Aug 2;9:74. doi: 10.1186/s13041-016-0256-9 (PMC4971702; doi:10.1186/s13041-016-0256-9)

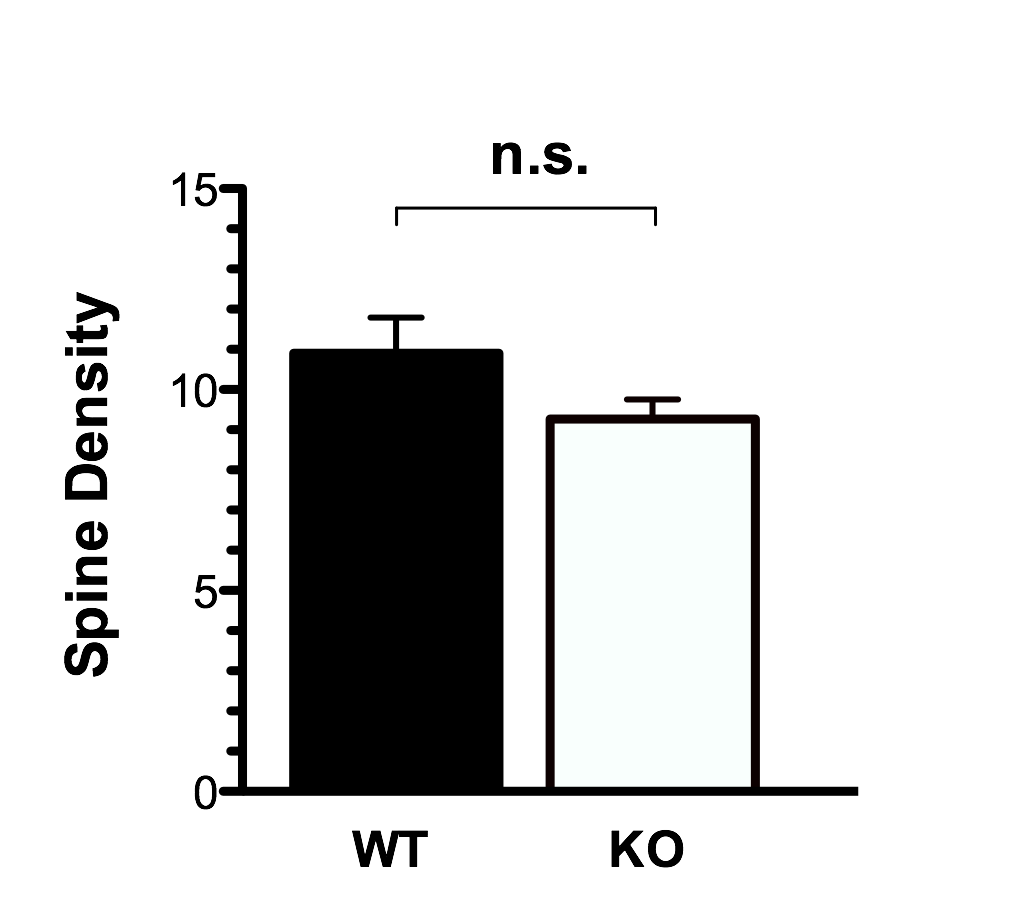

Supplement: Additional file 1: Figure S1. — Quantification of spine density between WT and Fmr1 KO hippocampal neurons. Fmr1 KO neurons display a decrease in spine density compared to WT neurons; although no significant statistical differences were detected (p > 0.05). (TIFF 3706 kb) [file 13041_2016_256_MOESM1_ESM.tiff]
